# Supplementary material for: Substrate recognition and cleavage by mucin degrading O-glycopeptidases from the gut microbe Bacteroides caccae
Source: J Biol Chem. 2026 Jun 4;302(8):113222. doi: 10.1016/j.jbc.2026.113222 (PMC13355702; doi:10.1016/j.jbc.2026.113222)
Supplement: Supporting Figures and Tables [file mmc2.pdf]

Supplementary Material for:

Substrate recognition and cleavage by mucin degrading *O*-glycopeptidases from *Bacteroides caccae*.

Benjamin Pluvinage[1,4], Kathleen Bourdon[1,4], Olivia Canil[1,4], Ashley Deventer[1,3], Bernadette Alvarez[1], Liam Mihalynuk[1], Nicole Thompson[2], Warren Wakarchuk[2], and Alisdair B. Boraston[1]*.

[1]Department of Biochemistry and Microbiology, University of Victoria, PO Box 1700 STN CSC, Victoria, British Columbia, V8W 2Y2, Canada.
[2]Department of Biological Sciences, University of Alberta, Edmonton, AB T6G 2E9, Canada.
[3]Current affiliation: Biomedical Sciences Research Complex, School of Biology, University of St Andrews, St Andrews, UK
[4]These authors contributed equally.

Running title: *B. caccae O*-glycopeptidases

*To whom correspondence should be addressed: Alisdair B. Boraston, Department of Biochemistry and Microbiology, University of Victoria, Victoria, British Columbia, Canada, V8P 5C2; boraston@uvic.ca; Tel. +1 (250) 472-4168; Fax. +1 (250) 721-8855

Supplementary Table 1. Linker sequences in FRET substrates and synthetic DNA fragments used to clone them. Green nucleotide indicates the 3’ region of the mNeonGreen domain, red the 5’ region of mScarletRed 1, and black the linker coding sequence.

Linker	Sequence	DNA Fragment
MUC1_R1	PAPGSTAPPAH	CCGATGGCCGCAAACTACCTTAAGAACCAGCCCATGTACGTGTTTCGGAAAACTGAGCTTAAACACAGCAAGACAGAGTTAAATTTCAAGGAGTGGCAAAAGGC<br>ATTCACCGACGTTAAGCCTGCACCGGGAAGCACGGCTCCCCCTGCTCATGTGAGCAAAGGTGAGGCCGTGATCAAGGAATTCATGCGTTTCAAGGTCC<br>ACATGGAAGGATCAATGAACGGACACGAATTCGAGATAGAAGGGGAAGGCGAAGGTCGTCCATACGAAGGTACGCAAACAGCAAAACTGAAGGTGACG
MUC1_R2	AHGVTSAPDTR	CCGATGGCCGCAAACTACCTTAAGAACCAGCCCATGTACGTGTTTCGGAAAACTGAGCTTAAACACAGCAAGACAGAGTTAAATTTCAAGGAGTGGCAAAAGGC<br>ATTCACCGACGTTAAGGCGCATGGGGTGACCTCAGCCCCTGACACACGTGTGAGCAAAGGTGAGGCCGTGATCAAGGAATTCATGCGTTTCAAGGTCC<br>ACATGGAAGGATCAATGAACGGACACGAATTCGAGATAGAAGGGGAAGGCGAAGGTCGTCCATACGAAGGTACGCAAACAGCAAAACTGAAGGTGACG
MUC2_R1	TTVTPTPTPTG	CCGATGGCCGCAAACTACCTTAAGAACCAGCCCATGTACGTGTTTCGGAAAACTGAGCTTAAACACAGCAAGACAGAGTTAAATTTCAAGGAGTGGCAAAAGGC<br>ATTCACCGACGTTAAGACCACGGTTACGCCTACCCCGACGCCTACTGGCGTGAGCAAAGGTGAGGCCGTGATCAAGGAATTCATGCGTTTCAAGGTCC<br>ACATGGAAGGATCAATGAACGGACACGAATTCGAGATAGAAGGGGAAGGCGAAGGTCGTCCATACGAAGGTACGCAAACAGCAAAACTGAAGGTGACG
MUC2_R2	VPPTTTPSPPP	CCGATGGCCGCAAACTACCTTAAGAACCAGCCCATGTACGTGTTTCGGAAAACTGAGCTTAAACACAGCAAGACAGAGTTAAATTTCAAGGAGTGGCAAAAGGC<br>ATTCACCGACGTTAAGGTCCCCCCAACTACAACTCCCTCTCCCCCGCCGGTGAGCAAAGGTGAGGCCGTGATCAAGGAATTCATGCGTTTCAAGGTCC<br>ACATGGAAGGATCAATGAACGGACACGAATTCGAGATAGAAGGGGAAGGCGAAGGTCGTCCATACGAAGGTACGCAAACAGCAAAACTGAAGGTGACG
MUC2_R3	SPPPTSTTTLP	CCGATGGCCGCAAACTACCTTAAGAACCAGCCCATGTACGTGTTTCGGAAAACTGAGCTTAAACACAGCAAGACAGAGTTAAATTTCAAGGAGTGGCAAAAGGC<br>ATTCACCGACGTTAAGTCACCACCGCCTACATCAACAACAACTCTGCCAGTGAGCAAAGGTGAGGCCGTGATCAAGGAATTCATGCGTTTCAAGGTCC<br>ACATGGAAGGATCAATGAACGGACACGAATTCGAGATAGAAGGGGAAGGCGAAGGTCGTCCATACGAAGGTACGCAAACAGCAAAACTGAAGGTGACG
MUC5AC_R1	PPTTSTTSAPP	CCGATGGCCGCAAACTACCTTAAGAACCAGCCCATGTACGTGTTTCGGAAAACTGAGCTTAAACACAGCAAGACAGAGTTAAATTTCAAGGAGTGGCAAAAGGC<br>ATTCACCGACGTTAAGCCGCCGACGACATCGACGACATCTGCTCCCCCCGTGAGCAAAGGTGAGGCCGTGATCAAGGAATTCATGCGTTTCAAGGTCC<br>ACATGGAAGGATCAATGAACGGACACGAATTCGAGATAGAAGGGGAAGGCGAAGGTCGTCCATACGAAGGTACGCAAACAGCAAAACTGAAGGTGACG
MUC5AC_R2	PVPATTVGPVP	CCGATGGCCGCAAACTACCTTAAGAACCAGCCCATGTACGTGTTTCGGAAAACTGAGCTTAAACACAGCAAGACAGAGTTAAATTTCAAGGAGTGGCAAAAGGC<br>ATTCACCGACGTTAAGCCCGTTCCCGCCACAACTGTTGGGCCTGTTCCTGTGAGCAAAGGTGAGGCCGTGATCAAGGAATTCATGCGTTTCAAGGTCC<br>ACATGGAAGGATCAATGAACGGACACGAATTCGAGATAGAAGGGGAAGGCGAAGGTCGTCCATACGAAGGTACGCAAACAGCAAAACTGAAGGTGACG
MUC5B_R1	GSTATPSSTPG	CCGATGGCCGCAAACTACCTTAAGAACCAGCCCATGTACGTGTTTCGGAAAACTGAGCTTAAACACAGCAAGACAGAGTTAAATTTCAAGGAGTGGCAAAAGGC<br>ATTCACCGACGTTAAGGGATCGACCGCCACACCGTCTTCAACACCTGGGGTGAGCAAAGGTGAGGCCGTGATCAAGGAATTCATGCGTTTCAAGGTCC<br>ACATGGAAGGATCAATGAACGGACACGAATTCGAGATAGAAGGGGAAGGCGAAGGTCGTCCATACGAAGGTACGCAAACAGCAAAACTGAAGGTGACG
MUC5B_R2	VLTTTATTPTP	CCGATGGCCGCAAACTACCTTAAGAACCAGCCCATGTACGTGTTTCGGAAAACTGAGCTTAAACACAGCAAGACAGAGTTAAATTTCAAGGAGTGGCAAAAGGC<br>ATTCACCGACGTTAAGGTACTTACTACTACGGCGACGACACCAACCCCTGTGAGCAAAGGTGAGGCCGTGATCAAGGAATTCATGCGTTTCAAGGTCC<br>ACATGGAAGGATCAATGAACGGACACGAATTCGAGATAGAAGGGGAAGGCGAAGGTCGTCCATACGAAGGTACGCAAACAGCAAAACTGAAGGTGACG
IgA1_h	SPSTPPTPSPS	CCGATGGCCGCAAACTACCTTAAGAACCAGCCCATGTACGTGTTTCGGAAAACTGAGCTTAAACACAGCAAGACAGAGTTAAATTTCAAGGAGTGGCAAAAGGC<br>ATTCACCGACGTTAAGTCACCGAGTACACCTCCTACACCGAGCCCTTCGGTGAGCAAAGGTGAGGCCGTGATCAAGGAATTCATGCGTTTCAAGGTCC<br>ACATGGAAGGATCAATGAACGGACACGAATTCGAGATAGAAGGGGAAGGCGAAGGTCGTCCATACGAAGGTACGCAAACAGCAAAACTGAAGGTGACG
MUC4	STGHATPLPVT	CCGATGGCCGCAAACTACCTTAAGAACCAGCCCATGTACGTGTTTCGGAAAACTGAGCTTAAACACAGCAAGACAGAGTTAAATTTCAAGGAGTGGCAAAAGGC<br>ATTCACCGACGTTAAGTCGACAGGCCATGCAACACCCCTTCCAGTAACAGTGAGCAAAGGTGAGGCCGTGATCAAGGAATTCATGCGTTTCAAGGTCC<br>ACATGGAAGGATCAATGAACGGACACGAATTCGAGATAGAAGGGGAAGGCGAAGGTCGTCCATACGAAGGTACGCAAACAGCAAAACTGAAGGTGACG
MUC3A/B	TSSITTTETTS	CCGATGGCCGCAAACTACCTTAAGAACCAGCCCATGTACGTGTTTCGGAAAACTGAGCTTAAACACAGCAAGACAGAGTTAAATTTCAAGGAGTGGCAAAAGGC<br>ATTCACCGACGTTAAGACAAGCTCGATTACCACAACTGAGACGACATCGGTGAGCAAAGGTGAGGCCGTGATCAAGGAATTCATGCGTTTCAAGGTCC<br>ACATGGAAGGATCAATGAACGGACACGAATTCGAGATAGAAGGGGAAGGCGAAGGTCGTCCATACGAAGGTACGCAAACAGCAAAACTGAAGGTGACG
MUC7_R1	TTAAPPTPSAT	CCGATGGCCGCAAACTACCTTAAGAACCAGCCCATGTACGTGTTTCGGAAAACTGAGCTTAAACACAGCAAGACAGAGTTAAATTTCAAGGAGTGGCAAAAGGC<br>ATTCACCGACGTTAAGACGACCGCTGCCCCCCCGACACCGTCGGCAACAGTGAGCAAAGGTGAGGCCGTGATCAAGGAATTCATGCGTTTCAAGGTCC<br>ACATGGAAGGATCAATGAACGGACACGAATTCGAGATAGAAGGGGAAGGCGAAGGTCGTCCATACGAAGGTACGCAAACAGCAAAACTGAAGGTGACG
MUC7_R2	PSATTPAPPSS	CCGATGGCCGCAAACTACCTTAAGAACCAGCCCATGTACGTGTTTCGGAAAACTGAGCTTAAACACAGCAAGACAGAGTTAAATTTCAAGGAGTGGCAAAAGGC<br>ATTCACCGACGTTAAGCCTTCAGCAACTACACCGGCCCCACCTTCGTCTGTGAGCAAAGGTGAGGCCGTGATCAAGGAATTCATGCGTTTCAAGGTCC<br>ACATGGAAGGATCAATGAACGGACACGAATTCGAGATAGAAGGGGAAGGCGAAGGTCGTCCATACGAAGGTACGCAAACAGCAAAACTGAAGGTGACG
MUC8_S1	PLQEGTPGSRV	CCGATGGCCGCAAACTACCTTAAGAACCAGCCCATGTACGTGTTTCGGAAAACTGAGCTTAAACACAGCAAGACAGAGTTAAATTTCAAGGAGTGGCAAAAGGC<br>ATTCACCGACGTTAAGCCATTGCAAGAAGGCACACCTGGATCTCGTGTGGTGAGCAAAGGTGAGGCCGTGATCAAGGAATTCATGCGTTTCAAGGTCC<br>ACATGGAAGGATCAATGAACGGACACGAATTCGAGATAGAAGGGGAAGGCGAAGGTCGTCCATACGAAGGTACGCAAACAGCAAAACTGAAGGTGACG
MUC8_S2	VHELPTSSPGG	CCGATGGCCGCAAACTACCTTAAGAACCAGCCCATGTACGTGTTTCGGAAAACTGAGCTTAAACACAGCAAGACAGAGTTAAATTTCAAGGAGTGGCAAAAGGC<br>ATTCACCGACGTTAAGGTGCACGAGTTGCCGACCAGCTCCCCTGGTGGCGTGAGCAAAGGTGAGGCCGTGATCAAGGAATTCATGCGTTTCAAGGTCC<br>ACATGGAAGGATCAATGAACGGACACGAATTCGAGATAGAAGGGGAAGGCGAAGGTCGTCCATACGAAGGTACGCAAACAGCAAAACTGAAGGTGACG
MUC10	TTDSTTPAPTT	CCGATGGCCGCAAACTACCTTAAGAACCAGCCCATGTACGTGTTTCGGAAAACTGAGCTTAAACACAGCAAGACAGAGTTAAATTTCAAGGAGTGGCAAAAGGC<br>ATTCACCGACGTTAAGACTACTGATTCCACCACGCCTGCGCCAACTACAGTGAGCAAAGGTGAGGCCGTGATCAAGGAATTCATGCGTTTCAAGGTCC<br>ACATGGAAGGATCAATGAACGGACACGAATTCGAGATAGAAGGGGAAGGCGAAGGTCGTCCATACGAAGGTACGCAAACAGCAAAACTGAAGGTGACG
MUC11/12	SSPGSTHTTLS	CCGATGGCCGCAAACTACCTTAAGAACCAGCCCATGTACGTGTTTCGGAAAACTGAGCTTAAACACAGCAAGACAGAGTTAAATTTCAAGGAGTGGCAAAAGGC<br>ATTCACCGACGTTAAGTCCTCCCCCGGATCTACTCACACTACACTGTCAGTGAGCAAAGGTGAGGCCGTGATCAAGGAATTCATGCGTTTCAAGGTCC<br>ACATGGAAGGATCAATGAACGGACACGAATTCGAGATAGAAGGGGAAGGCGAAGGTCGTCCATACGAAGGTACGCAAACAGCAAAACTGAAGGTGACG
MUC16	SVPTTSTPGTS	CCGATGGCCGCAAACTACCTTAAGAACCAGCCCATGTACGTGTTTCGGAAAACTGAGCTTAAACACAGCAAGACAGAGTTAAATTTCAAGGAGTGGCAAAAGGC<br>ATTCACCGACGTTAAGAGTGTGCCGACAACCTCGACACCAGGGACAAGCGTGAGCAAAGGTGAGGCCGTGATCAAGGAATTCATGCGTTTCAAGGTCC<br>ACATGGAAGGATCAATGAACGGACACGAATTCGAGATAGAAGGGGAAGGCGAAGGTCGTCCATACGAAGGTACGCAAACAGCAAAACTGAAGGTGACG
MUC19	STTVAPGSTTV	CCGATGGCCGCAAACTACCTTAAGAACCAGCCCATGTACGTGTTTCGGAAAACTGAGCTTAAACACAGCAAGACAGAGTTAAATTTCAAGGAGTGGCAAAAGGC<br>ATTCACCGACGTTAAGTCGACTACTGTTGCTCCAGGTAGCACAACGGTAGTGAGCAAAGGTGAGGCCGTGATCAAGGAATTCATGCGTTTCAAGGTCC<br>ACATGGAAGGATCAATGAACGGACACGAATTCGAGATAGAAGGGGAAGGCGAAGGTCGTCCATACGAAGGTACGCAAACAGCAAAACTGAAGGTGACG
ASF	AEAPTAVPDKG	CCGATGGCCGCAAACTACCTTAAGAACCAGCCCATGTACGTGTTTCGGAAAACTGAGCTTAAACACAGCAAGACAGAGTTAAATTTCAAGGAGTGGCAAAAGGC<br>ATTCACCGACGTTAAGGCAGAGGCCCCGACTGCCGTGCCTGATAAAGGCGTGAGCAAAGGTGAGGCCGTGATCAAGGAATTCATGCGTTTCAAGGTCC<br>ACATGGAAGGATCAATGAACGGACACGAATTCGAGATAGAAGGGGAAGGCGAAGGTCGTCCATACGAAGGTACGCAAACAGCAAAACTGAAGGTGACG
Glycoprotein Iba	PAPSPTTPEPT	CCGATGGCCGCAAACTACCTTAAGAACCAGCCCATGTACGTGTTTCGGAAAACTGAGCTTAAACACAGCAAGACAGAGTTAAATTTCAAGGAGTGGCAAAAGGC<br>ATTCACCGACGTTAAGCCGGCACCCTCACCCACTACTCCGGAGCCAACTGTGAGCAAAGGTGAGGCCGTGATCAAGGAATTCATGCGTTTCAAGGTCC<br>ACATGGAAGGATCAATGAACGGACACGAATTCGAGATAGAAGGGGAAGGCGAAGGTCGTCCATACGAAGGTACGCAAACAGCAAAACTGAAGGTGACG
MUC6_R1	TTTYPTPSHPQ	CCGATGGCCGCAAACTACCTTAAGAACCAGCCCATGTACGTGTTTCGGAAAACTGAGCTTAAACACAGCAAGACAGAGTTAAATTTCAAGGAGTGGCAAAAGGC<br>ATTCACCGACGTTAAGACGACGACGTATCCCACCCCGAGTCACCCTCAAGTGAGCAAAGGTGAGGCCGTGATCAAGGAATTCATGCGTTTCAAGGTCC<br>ACATGGAAGGATCAATGAACGGACACGAATTCGAGATAGAAGGGGAAGGCGAAGGTCGTCCATACGAAGGTACGCAAACAGCAAAACTGAAGGTGACG
MUC17_R1	SPTNSSPTTAE	CCGATGGCCGCAAACTACCTTAAGAACCAGCCCATGTACGTGTTTCGGAAAACTGAGCTTAAACACAGCAAGACAGAGTTAAATTTCAAGGAGTGGCAAAAGGC<br>ATTCACCGACGTTAAGTCACCAACCAATTCGTCTCCGACCACTGCTGAGGTGAGCAAAGGTGAGGCCGTGATCAAGGAATTCATGCGTTTCAAGGTCC<br>ACATGGAAGGATCAATGAACGGACACGAATTCGAGATAGAAGGGGAAGGCGAAGGTCGTCCATACGAAGGTACGCAAACAGCAAAACTGAAGGTGACG
Primer 1		TTTGCGGCCATCGGCTTAGC
Primer 2		AAACTGAAGGTGACGAAAGGTGGCC

Supplementary Table 2. Properties of *Bacteroides caccae* M60 peptidases.

B. caccae M60	Locus Tag (new assembly)	Locus Tag (original assembly)	Molecular Weight (g/mol)	Extinction Coefficient (M^{-1} cm^{-1})	*E. coli* expression strain
BcM60A	CGC64_02005	BACCAC_00161	61278.36	107635	BL21 (DE3)
BcM60B	CGC64_02920	BACCAC_01246	53885.47	79565	BL21 (DE3)
BcM60C	CGC64_03500	BACCAC_01368	59409.12	112565	BL21 (DE3)
BcM60D	CGC64_03540	BACCAC_01376	61326.58	92750	Shuffle T7
BcM60E	CGC64_03545	BACCAC_01377	61439.74	117730	BL21 (DE3)
BcM60F	CGC64_04090	BACCAC_01495	59371.78	87920	BL21 (DE3)
BcM60G	CGC64_04095	BACCAC_01496	49739.70	84355	BL21 (DE3)
BcM60H	CGC64_07210	BACCAC_01782	62521.22	127130	BL21 (DE3)
BcM60I	CGC64_07485	BACCAC_01840	64460.95	118065	Shuffle T7
BcM60J	CGC64_07490	BACCAC_01841	65174.52	138910	Shuffle T7
BcM60K	CGC64_07580	BACCAC_01859	60926.43	97555	BL21 (DE3)
BcM60L	CGC64_08375	BACCAC_03612	60932.89	107065	BL21 (DE3)
BcM60M	CGC64_07535	BACCAC_01850			BL21 (DE3)
BcM60N	CGC64_14820	BACCAC_02574	N/A	N/A	N/A
BcM60O	CGC64_14820	BACCAC_02574	N/A	N/A	N/A
BcM60P	CGC64_16100	BACCAC_02897	N/A	N/A	N/A

Supplementary Table 3. Crystallization conditions of *Bacteroides caccae* M60 peptidases.

Protein	Notes	[Protein]	Crystallization condition	Cryoprotectant
BcM60B	His tag removed	12.5	4% Tascimate pH4.0<br>15% PEG3350<br>Cryo 20% EG	20% ethylene glycol
BcM60C<br>WT and<br>E665Q mutant	His tag removed	29	0.2M $(NH_4)_2\ SO_4$<br>0.1M Na citrate pH5.6<br>21% PEG4000	20% ethylene glycol
BcM60F	His tag removed	10	0.1M Na citrate tribasic dihydrate pH5.6<br>20% 2-Propanol<br>20% PEG4K	20% ethylene glycol
BcM60K<br>WT and E664A mutant	His tag removed	15.3	0.1M Bis-Tris pH6.0<br>20% PEG3350	20% ethylene glycol

Supplementary Table 4. X-ray Data collection and structure statistics. Values for highest resolution shells are shown in parenthesis.

	BcM60K	BcM60K C2	BcM60K 6SC1
Data Collection			
Beamline	Home Beam	Home Beam	Home Beam
Wavelength (Å)	1.54178	1.54178	1.54178
Space Group	$P2_1$	$P2_1$	$P2_1$
Cell Dimensions			
a, b, c (Å)	46.9, 64.9, 93.4	46.9, 64.9, 93.6	46.5, 65.1, 93.2
α, β, γ (º)	90.0, 100.8, 90.0	90.0, 100.8, 90.0	90.0, 100.6, 90.0
Resolution (Å)	30.00-2.10 (2.14-2.10)	30.00-2.50 (2.54-2.50)	30.00-2.50 (2.54-2.50)
R_{meas}	0.092 (0.288)	0.144 (0.709)	0.078 (0.289)
R_{pim}	0.047 (0.199)	0.071 (0.410)	0.042 (0.183)
CC1/2	0.983 (0.894)	0.987 (0.631)	0.994 (0.912)
$\langle I/\sigma I \rangle$	11.6 (2.5)	8.9 (1.7)	14.4 (3.3)
Completeness (%)	98.8 (94.2)	99.5 (95.2)	99.1 (92.8)
Redundancy	2.9 (2.3)	3.8 (2.6)	2.9 (2.0)
No. of reflections	92946	73443	53428
No. Unique	31868 (1503)	19286 (885)	19338 (883)
Refinement			
Resolution (Å)	2.10	2.5	2.5
R_{work}/R_{free}	0.22/0.25	0.02/0.24	0.22/0.28
No. of atoms			
Protein	4221	4187	4193
Ligand	1 (ZN), 28 (EDO), 14 (BTB)	1 (ZN), 47 (C2)	1 (ZN), 20 (EDO), 52 (6SC1)
Water	263	200	92
B-factors			
Protein	29.3	41.5	40.0
Ligand	29.9 (ZN), 30.0 (EDO), 46.9 (BTB)	44.1 (ZN), 50.1 (C2)	40.2 (ZN), 38.9 (EDO), 45.8 (6SC1)
Water	27.9	37.9	33.5
r.m.s.d			
Bond lengths (Å)	0.001	0.003	0.002
Bond angles (°)	0.416	0.539	0.469
Ramachandran (%)			
Preferred	94.8	95.6	94.5
Allowed	5.2	4.4	5.5
Disallowed	0.0	0.0	0.0

	BcM60K_E664A Muc5AC peptide	BcM60F	BcM60B
Data Collection			
Beamline	Home Beam	Home Beam	Home Beam
Wavelength (Å)	1.54178	1.54178	1.54178
Space Group	$P2_1$	P21	P21
Cell Dimensions			
a, b, c (Å)	46.9, 65.0, 93.6	78.3, 96.7, 85.9	48.1, 109.4, 96.8
α, β, γ (º)	90.0, 100.9, 90.0	90.0, 114.6, 90.0	90.0, 94.8, 90.0
Resolution (Å)	25.00-2.20 (2.24-2.20)	30.00-1.80 (1.83-1.80)	30.00-1.80 (1.83-1.80)
R_{meas}	0.106 (0.525)	0.055 (0.166)	0.070 (0.251)
R_{pim}	0.052 (0.290)	0.025 (0.100)	0.034 (0.162)
CC1/2	0.919 (0.997)	0.998 (0.959)	0.997 (0.906)
<I/σI>	13.1 (2.4)	27.4 (6.2)	19.4 (4.2)
Completeness (%)	99.6 (96.2)	99.0 (97.8)	100.0 (99.6)
Redundancy	3.8 (2.5)	3.9 (2.9)	4.0 (2.9)
No. of reflections	106679	413212	371777
No. Unique	28358 (1401)	106768 (5256)	92252 (4571)
Refinement			
Resolution (Å)	2.20	1.80	1.80
R_{work}/R_{free}	0.18/0.22	0.15/0.18	0.17/0.20
No. of atoms			
Protein	4254	4122 (A), 4104 (B)	3771 (A), 3829 (B)
Ligand	1 (ZN), 47 (peptide)	48 (EDO)	2 (ZN), 40 EDO), 21 (MLA), 1 (CA), 15 (FMT), 16 (SIN)
Water	407	1085	714
B-factors			
Protein		18.7 (A), 17.7 (B)	20.5 (A), 18.0 (B)
Ligand	35.4 (ZN), 59.6 (peptide)	29.2 (EDO)	28.5 (ZN), 25.8 (EDO),31.0 (MLA), 22.8 (CA), 27.6 (FMT), 33.9 (SIN)
Water	38.4	27.0	26.4
r.m.s.d			
Bond lengths (Å)	0.008	0.007	0.011
Bond angles (°)	0.870	0.902	1.756
Ramachandran (%)			
Preferred	95.0	97.7	97.9
Allowed	5.0	2.3	2.1
Disallowed	0.0	0.0	0.0

	BcM60C	BcM60C 6SC1	BcM60C_E665Q C2
Data Collection			
Beamline	Home Beam	Home Beam	Home Beam
Wavelength (Å)	1.54178	1.54178	1.54178
Space Group	$P4_12_12$	$P4_12_12$	$P4_12_12$
Cell Dimensions			
a, b, c (Å)	118.6, 118.6, 90.9	118.5, 118.5, 90.8	118.4, 118.4, 88.2
α, β, γ (°)	90.0, 90.0, 90.0	90.0, 90.0, 90.0	90.0, 90.0, 90.0
Resolution (Å)	30.00-1.55 (1.58-1.55)	30.00-1.55 (1.58-1.55)	25.00-2.15 (2.19-2.15)
R_{merge}	0.089 (0.569)	0.056 (0.384)	0.087 (0.422)
R_{pim}	0.026 (0.263)	0.022 (0.267)	0.039 (0.307)
CC1/2	0.997 (0.855)	0.997 (0.856)	0.998 (0.778)
<I/σI>	27.5 (2.4)	27.8 (2.3)	16.1 (2.1)
Completeness (%)	99.8 (99.9)	99.8 (99.7)	98.5 (87.1)
Redundancy	7.8 (5.0)	4.2 (2.7)	4.1 (2.1)
No. of reflections	730718	396537	140286
No. Unique	93992 (4620)	93659 (4620)	34134 (1451)
Refinement			
Resolution (Å)	1.55	1.55	2.15
R_{work}/R_{free}	0.18/0.21	0.18/0.21	0.21/0.26
No. of atoms			
Protein	4173	4182	4038
Ligand	1 (ZN), 15 (SO4), 16 (EDO)	1 (ZN), 15 (SO4), 16 (EDO), 52 (STAg)	1 (ZN), 10 (SO4), 24 (EDO), 47 (Thr_Core2)
Water	678	674	230
B-factors			
Protein	17.2	17.1	27.5
Ligand	16.6 (ZN), 37.9 (SO4), 28.4 (EDO),	15.0 (ZN), 28.2 (SO4), 28.7 (EDO), 32.3 (STAg)	24.4 (ZN), 45.2 (SO4), 39.6 (SO4), 35.8 (Thr_Core2)
Water	28.0	27.9	27.6
r.m.s.d			
Bond lengths (Å)	0.009	0.010	0.002
Bond angles (°)	1.051	1.081	0.453
Ramachandran (%)			
Preferred	96.9	97.1	95.3
Allowed	3.1	2.9	4.7
Disallowed	0.0	0.0	0.0

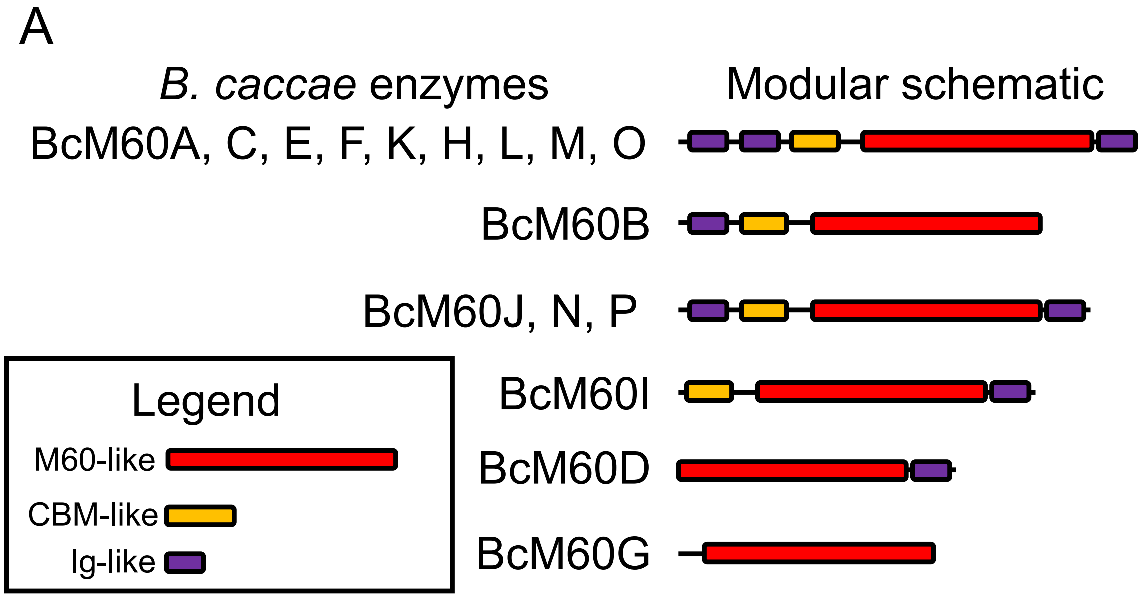
A
B. caccae enzymes
Modular schematic
BcM60A, C, E, F, K, H, L, M, O
BcM60B
BcM60J, N, P
BcM60I
BcM60D
BcM60G
Legend
M60-like
CBM-like
Ig-like

B

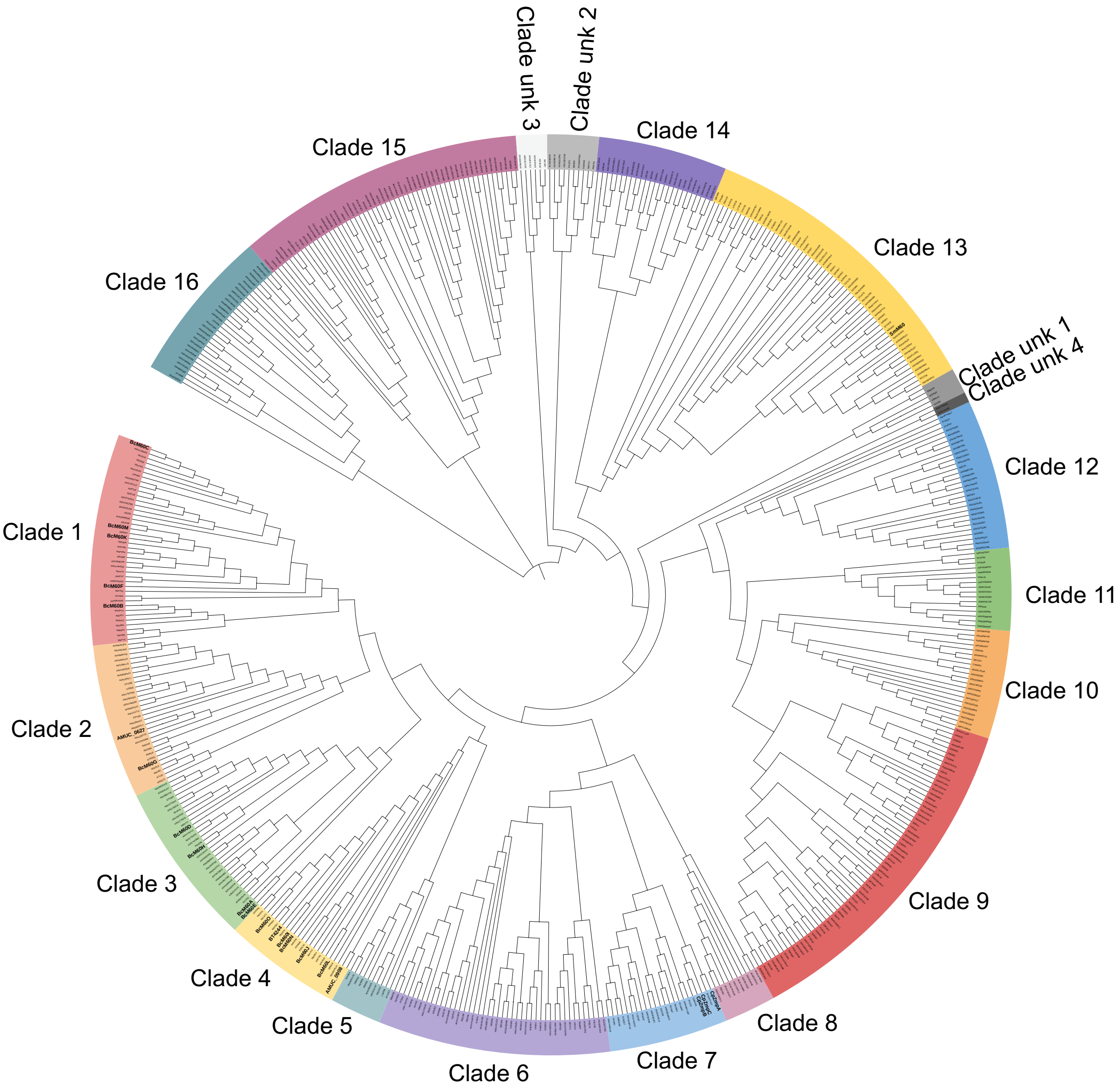

Supplementary Figure 1. A) Modular schematics of the predicted *B. caccae* peptidase_M60 proteins. B) Complete phylogenetic tree of peptidase_M60 proteins from Pfam entry 13402 (Pfam version 32). Clades were defined with a roughly 35% amino acid sequence identity cutoff. The *B. caccae* proteins and entries with prior characterization are labeled.

A

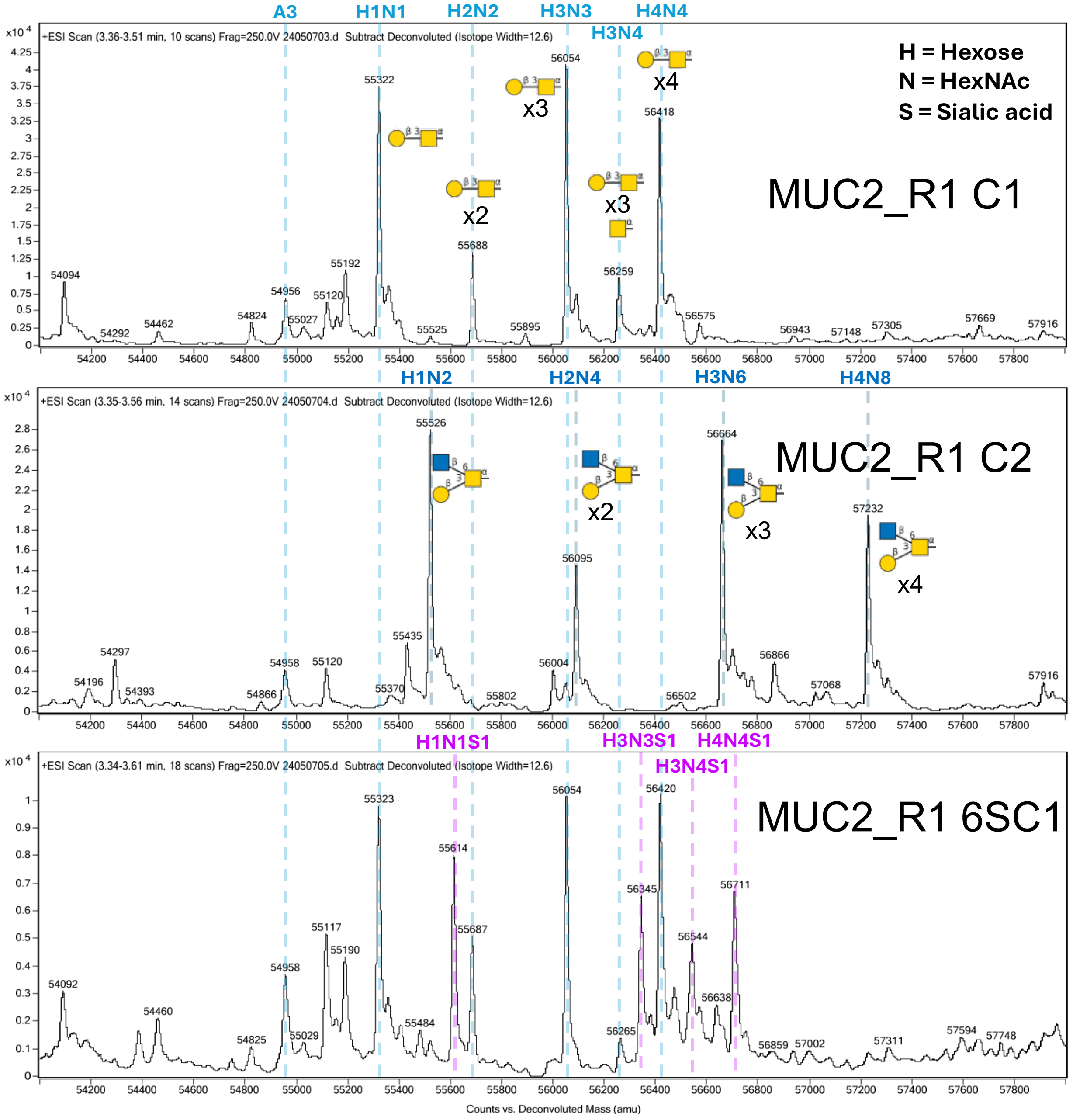
A3
H1N1
H2N2
H3N3
H4N4
H3N4
H = Hexose
N = HexNAc
S = Sialic acid
MUC2_R1 C1
+ESI Scan (3.36-3.51 min. 10 scans) Frag=250.0V 24050703.d Subtract Deconvoluted (Isotope Width=12.6)
x2
x3
x4
H1N2
H2N4
H3N6
H4N8
MUC2_R1 C2
+ESI Scan (3.35-3.56 min. 14 scans) Frag=250.0V 24050704.d Subtract Deconvoluted (Isotope Width=12.6)
H1N1S1
H3N3S1
H4N4S1
H3N4S1
MUC2_R1 6SC1
+ESI Scan (3.34-3.61 min. 18 scans) Frag=250.0V 24050705.d Subtract Deconvoluted (Isotope Width=12.6)
Counts vs. Deconvoluted Mass (amu)

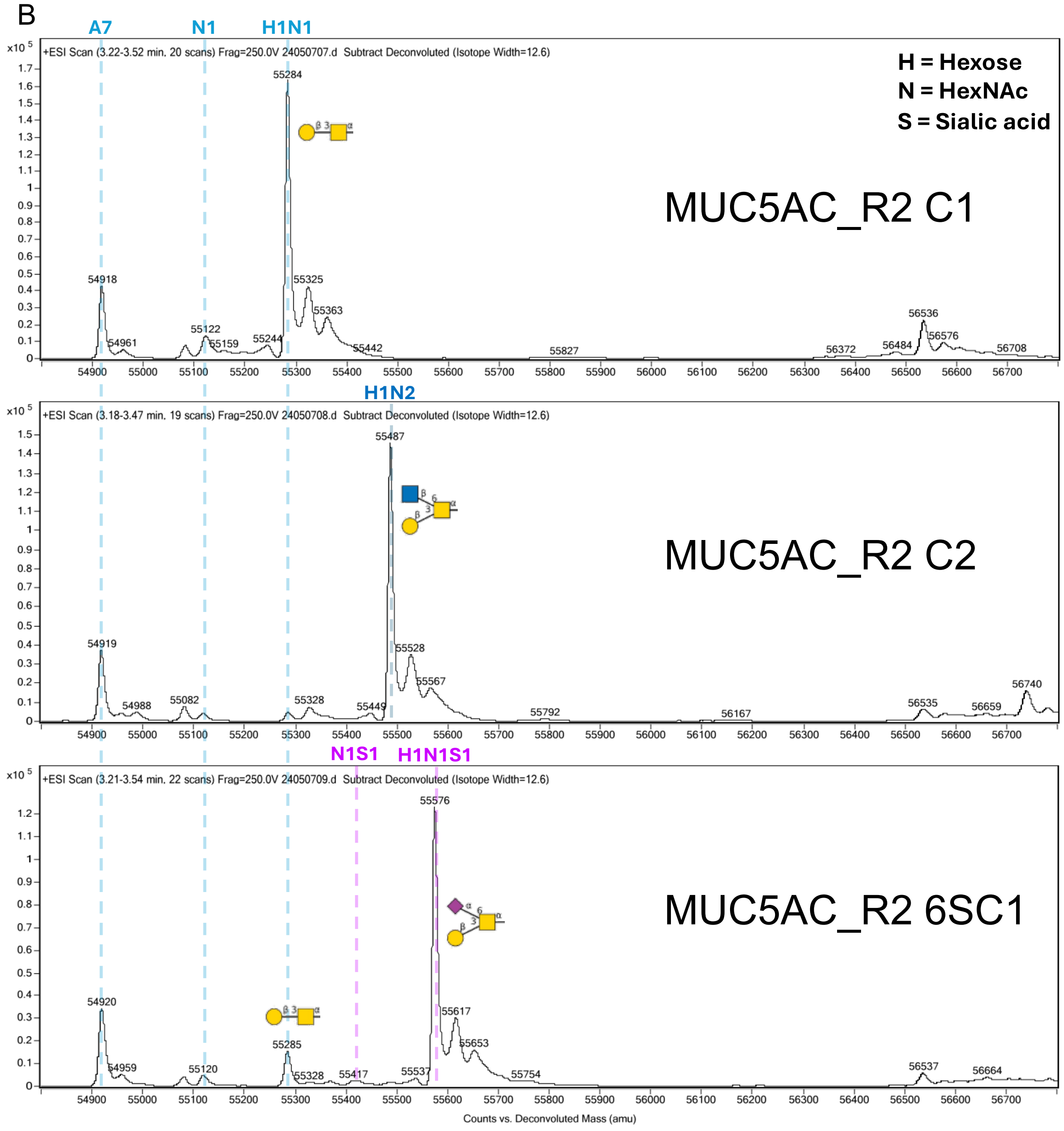
B
A7
N1
H1N1
H1N2
N1S1
H1N1S1
H = Hexose
N = HexNAc
S = Sialic acid
MUC5AC_R2 C1
MUC5AC_R2 C2
MUC5AC_R2 6SC1
+ESI Scan (3.22-3.52 min, 20 scans) Frag=250.0V 24050707.d Subtract Deconvoluted (Isotope Width=12.6)
+ESI Scan (3.18-3.47 min, 19 scans) Frag=250.0V 24050708.d Subtract Deconvoluted (Isotope Width=12.6)
+ESI Scan (3.21-3.54 min, 22 scans) Frag=250.0V 24050709.d Subtract Deconvoluted (Isotope Width=12.6)
x10 5
55284
54918
54961
55122
55159
55244
55325
55363
55442
55827
56372
56484
56536
56576
56708
55487
54919
54988
55082
55328
55449
55528
55567
55792
56167
56535
56659
56740
55576
54920
54959
55120
55285
55328
55417
55537
55617
55653
55754
56537
56664
Counts vs. Deconvoluted Mass (amu)

C

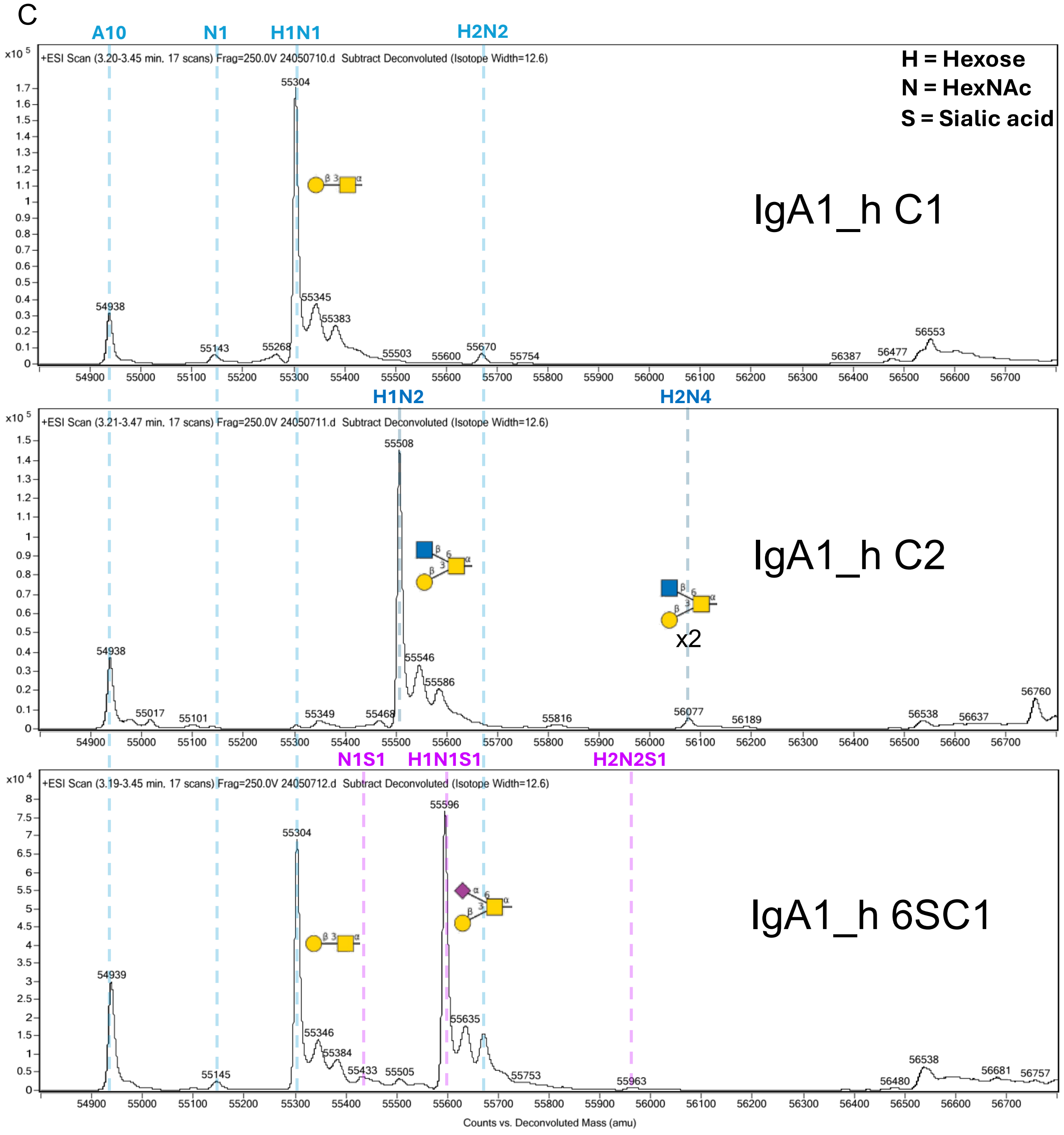
A10
N1
H1N1
H2N2
H = Hexose
N = HexNAc
S = Sialic acid
+ESI Scan (3.20-3.45 min, 17 scans) Frag=250.0V 24050710.d Subtract Deconvoluted (Isotope Width=12.6)
IgA1_h C1
55304
54938
55143
55268
55345
55383
55503
55600
55670
55754
56387
56477
56553
H1N2
H2N4
+ESI Scan (3.21-3.47 min, 17 scans) Frag=250.0V 24050711.d Subtract Deconvoluted (Isotope Width=12.6)
IgA1_h C2
55508
54938
55017
55101
55349
55468
55546
55586
55816
56077
56189
56538
56637
56760
x2
N1S1
H1N1S1
H2N2S1
+ESI Scan (3.19-3.45 min, 17 scans) Frag=250.0V 24050712.d Subtract Deconvoluted (Isotope Width=12.6)
IgA1_h 6SC1
55596
55304
54939
55145
55346
55384
55433
55505
55635
55753
55963
56480
56538
56681
56757
Counts vs. Deconvoluted Mass (amu)

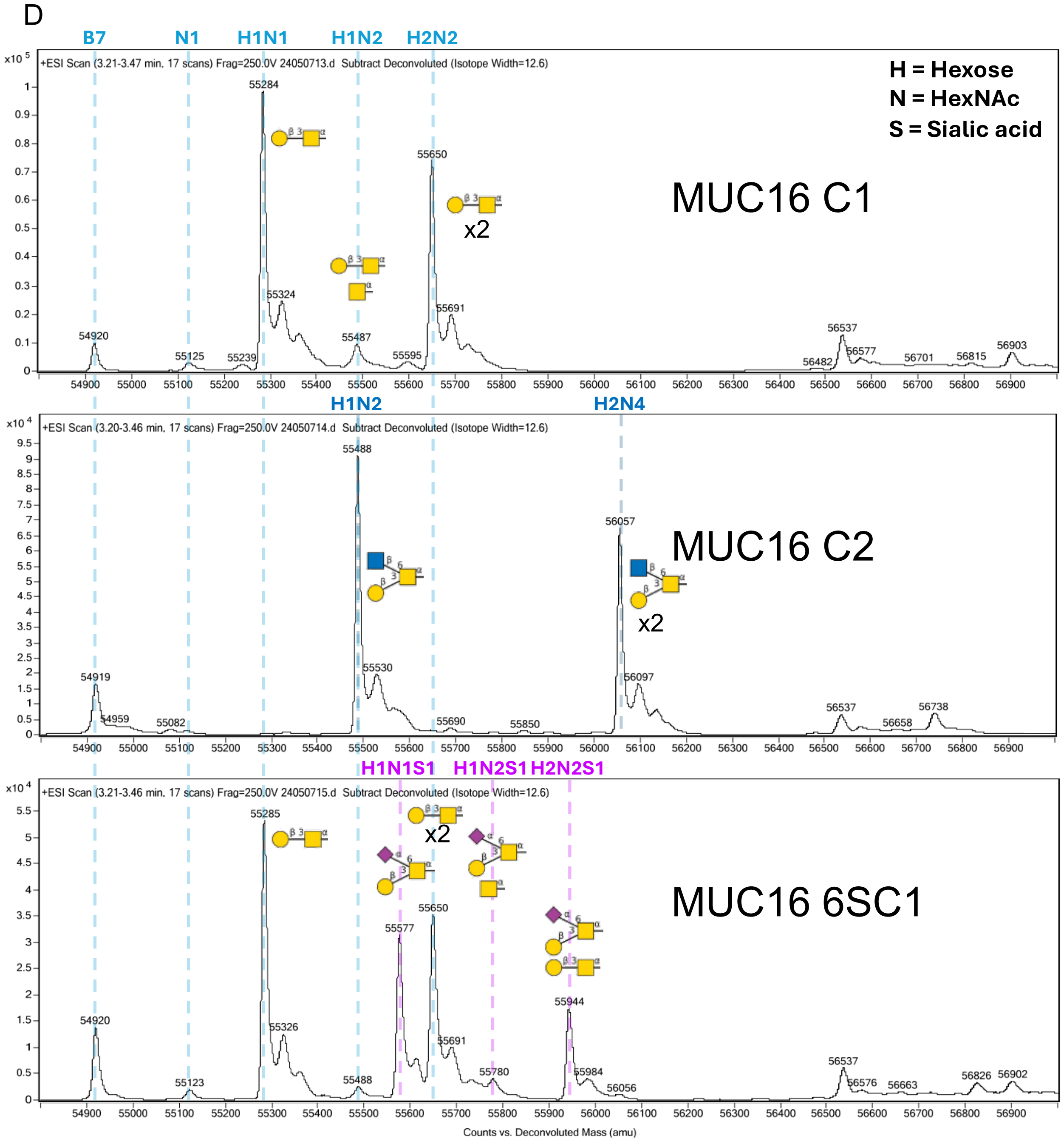

Supplementary Figure 2. Intact mass spectrometry analysis of four FRET substrates modified with C1, C2, and 6SC1. A) MUC2_R1 substrate. B) MUC5AC_R2 substrate. C) IgA1_h substrate. D) MUC16 substrate. Linker identities are provided in each panel.

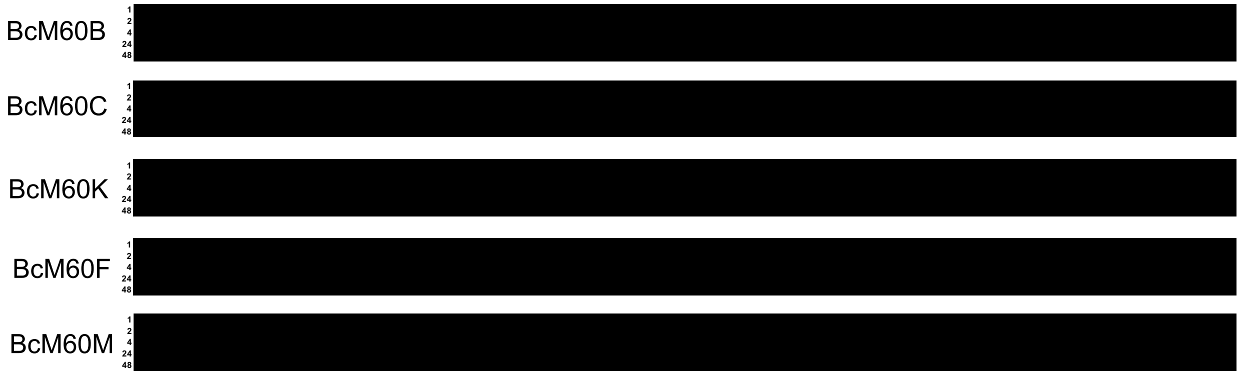

Supplementary Figure 3. FRET screens using unglycosylated substrates. Heat maps show FRET values calculated by the ratio-of-ratios approach. The coloring is ramped from a low threshold of 0.2 to a high of 1.0. Numbers just to the left of the heat maps indicate the time of incubation in hours. The observation that all of the samples are black indicates a lack of significant activity.

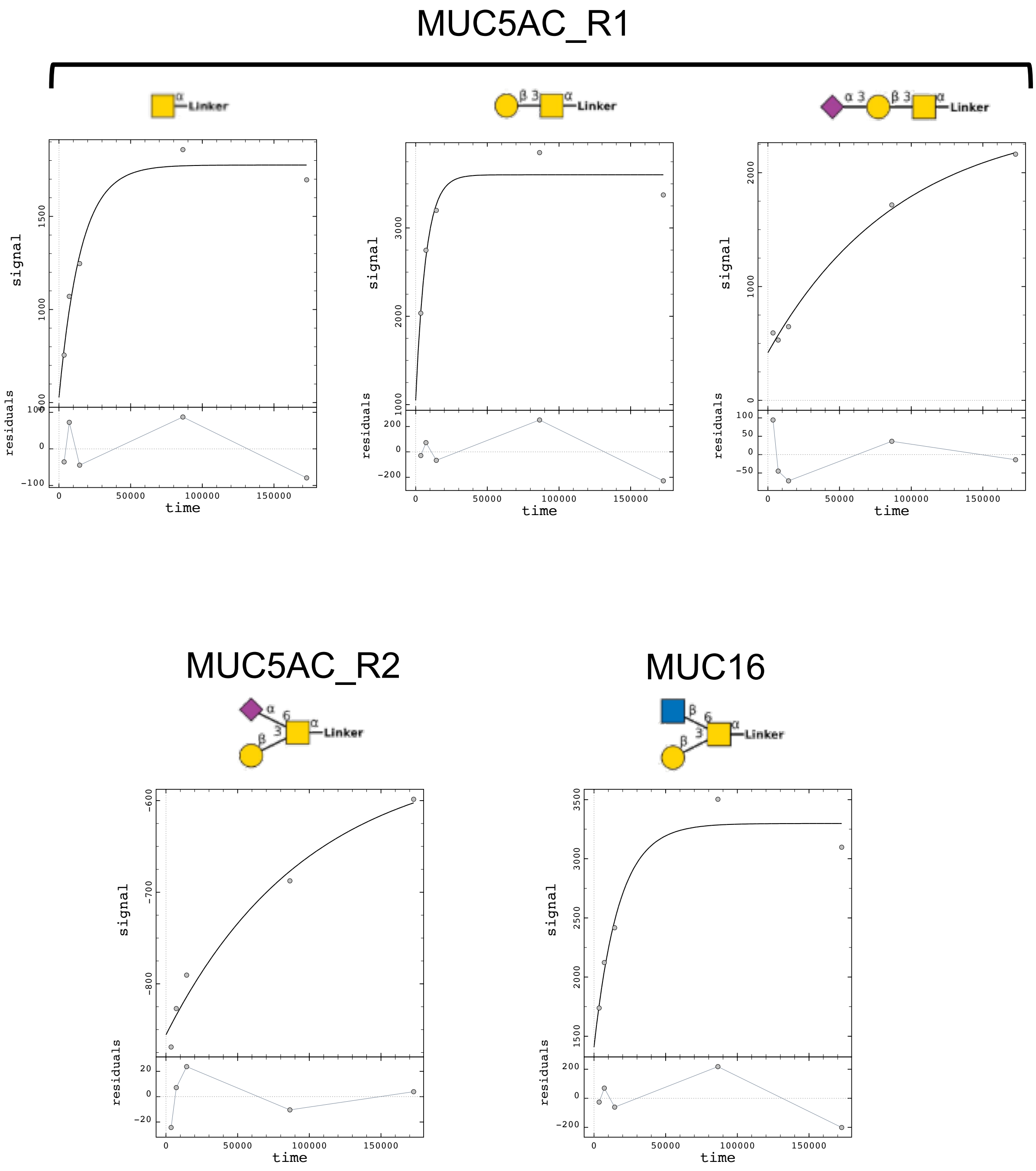

Supplementary Figure 4. Examples of FRET screen data converted to fluorescence difference values and fit to a simple bi-molecular kinetic model (*i.e.*, the so-called "hit-and-run" approach). All data shown is for BcM60K.

BcM60K
MUC5AC_R2 Tn

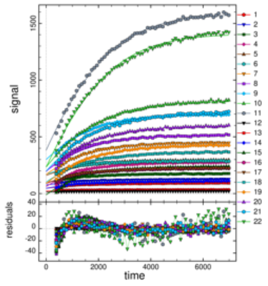

BcM60K
MUC5AC_R2 C1

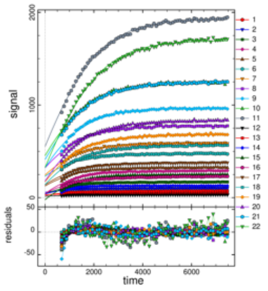

BcM60K
MUC5AC_R2 3SC1

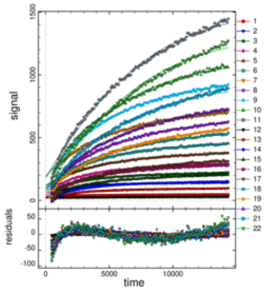

BcM60K
MUC5AC_R2 C2

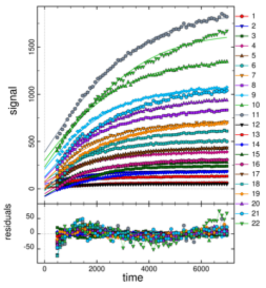

BcM60K
MUC5AC_R2 6SC1

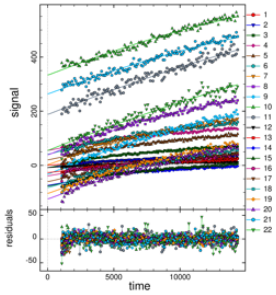

BcM60K
MUC5AC_R2 (repeat)

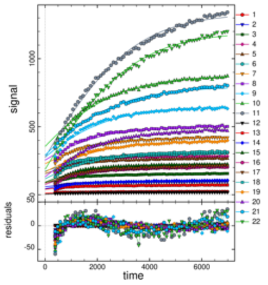

BcM60K
5AC_R2/11 hybrid

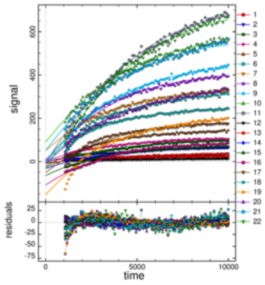

BcM60K
11/5AC_R2 hybrid

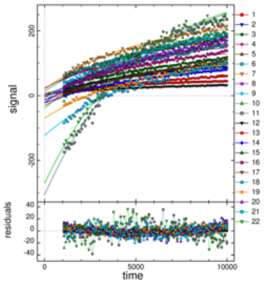

BcM60K
5AC_R2/IgA hybrid

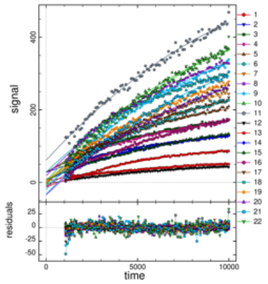

BcM60K
4/5AC_R2 hybrid

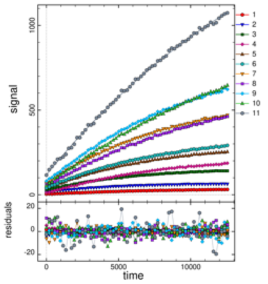

BcM60K
5AC_R2/4 hybrid

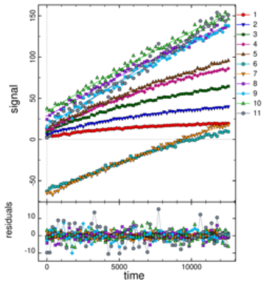

BcM60C
MUC5AC_R2 Tn

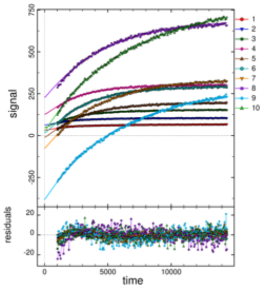

BcM60C
MUC5AC_R2 C1

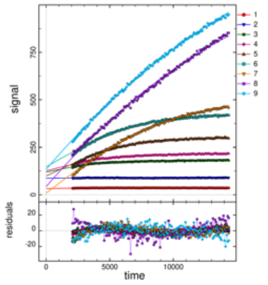

BcM60F
MUC5AC_R2 Tn

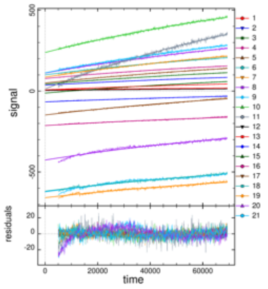

BcM60F
MUC5AC_R2 C1

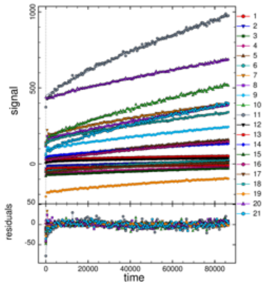

BcM60F
MUC5AC_R2 3SC1

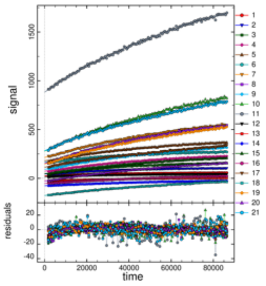

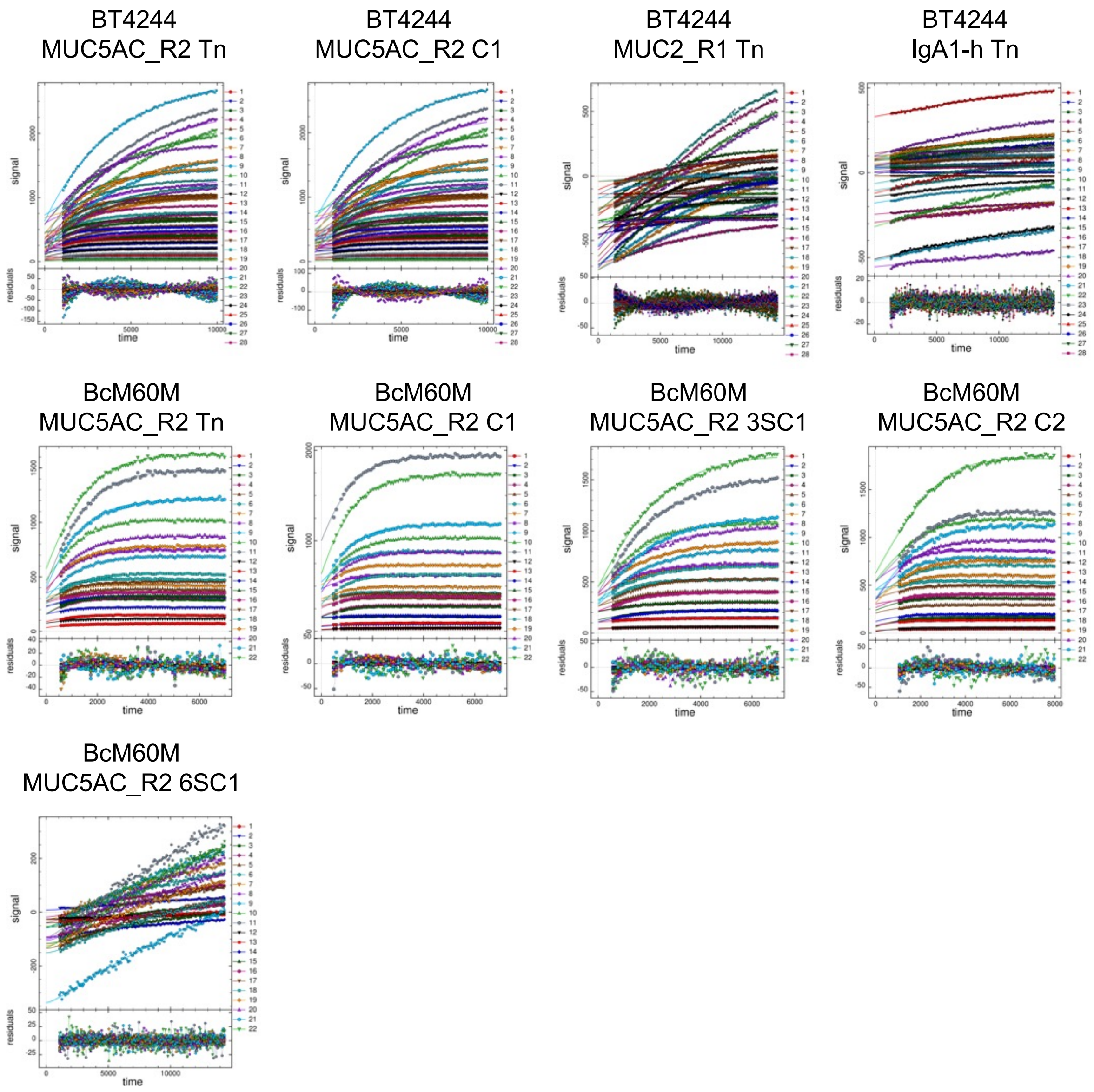

Supplementary Figure 5 (and preceding page). Enzyme progress curves used for deriving kinetic constants. The enzyme and substrate used are shown. Symbols indicate the experimental data and solid lines the fit to the model as described in the materials and methods.

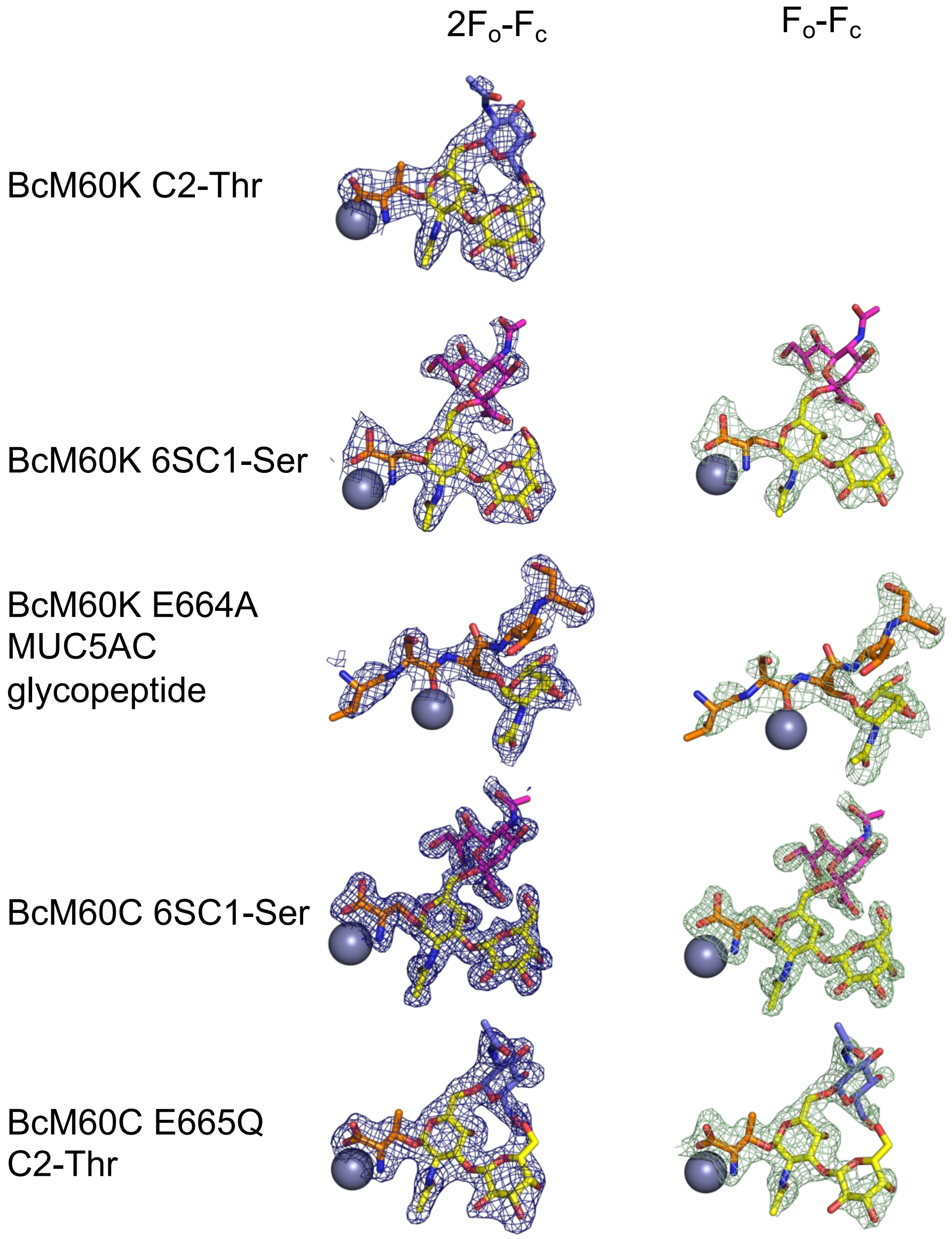

Supplementary Figure 6. Experimental electron density maps for ligands in the BcM60K and BcM60C complexes. $2F_o$-F_c electron density maps are contoured at 1σ and shown as blue mesh. F_o-F_c electron density maps generated by refinement in the absence of ligand are contoured at 3σ and shown as green mesh. The $2F_o$-F_c and F_o-F_c electron density maps for the BcM60K E664A peptide complex are shown contoured at 0.9σ and 2.5σ, respectively.

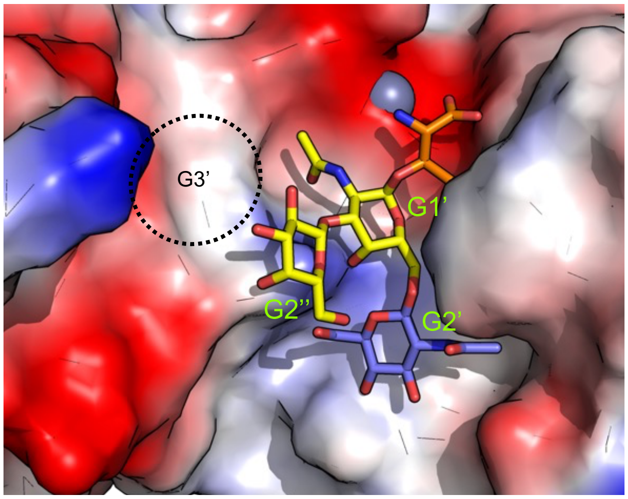

Supplementary Figure 7. The surface of the active site of a BcM60M model generated with AlphaFold 3. The C2-Thr was modeled in based on an overlap with the BcM60C C2-Thr complex.

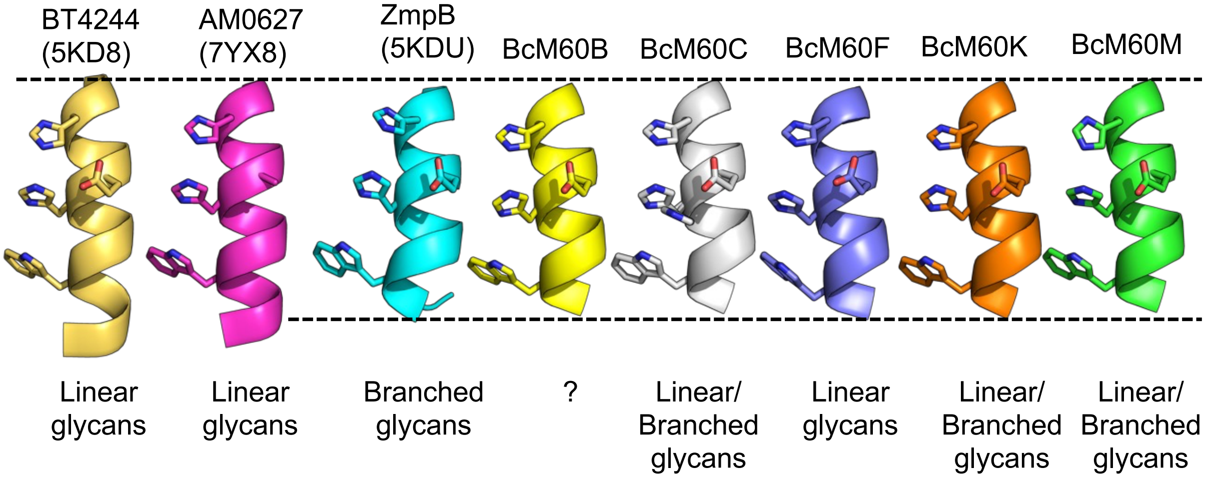

Supplementary Figure 8. Comparison of catalytic helix length in characterized peptidase_M60 O-glycopeptidases and in the new BcM60 enzymes. The zinc binding histidines, catalytic glutamate, and GalNAc binding tryptophan are shown for reference. AM0627 refers to AMUC_0627; this structure was an alanine mutant of the catalytic glutamate.

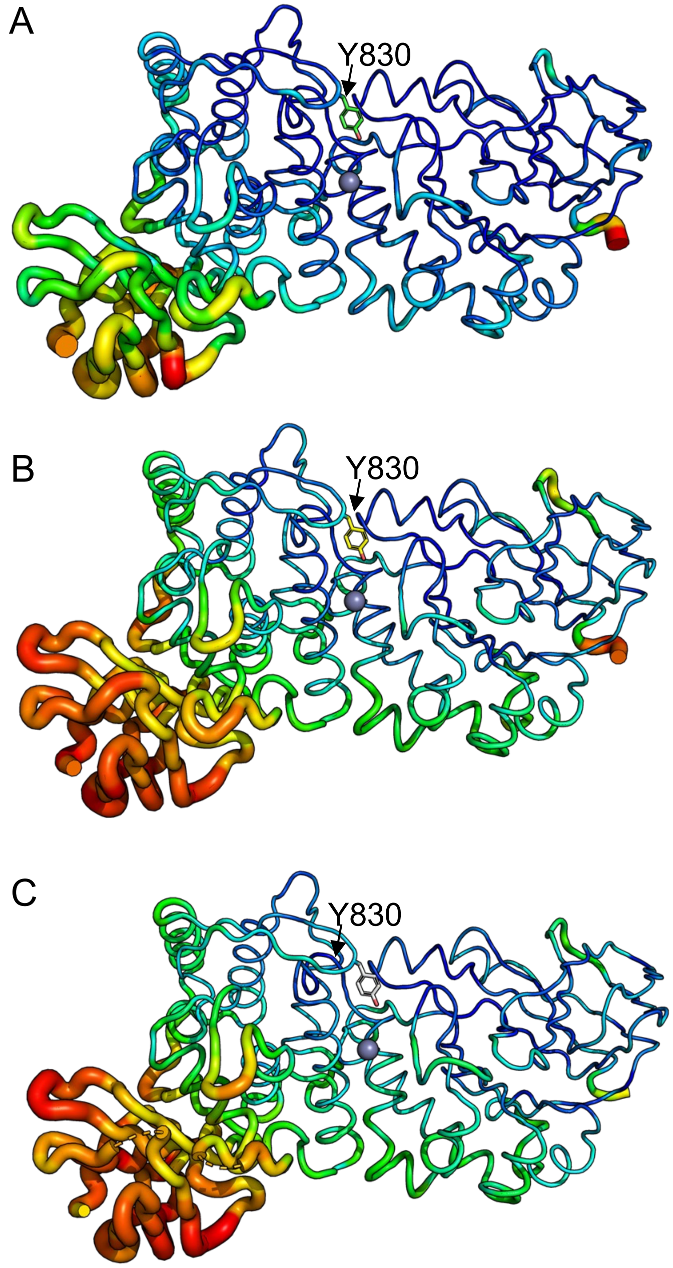

Supplementary Figure 9. Tube representations of BcM60K structures. A) complex of BcM60K E664A with the MUC5AC glycopeptide. B) Complex of BcM60K with 6SC1-Ser. C) Unliganded BcM60K. The thickness of the tube from thin to thick and color ramp from blue to red indicate increasing B-factors. The tyrosine residue closing in on the active site is shown as sticks.

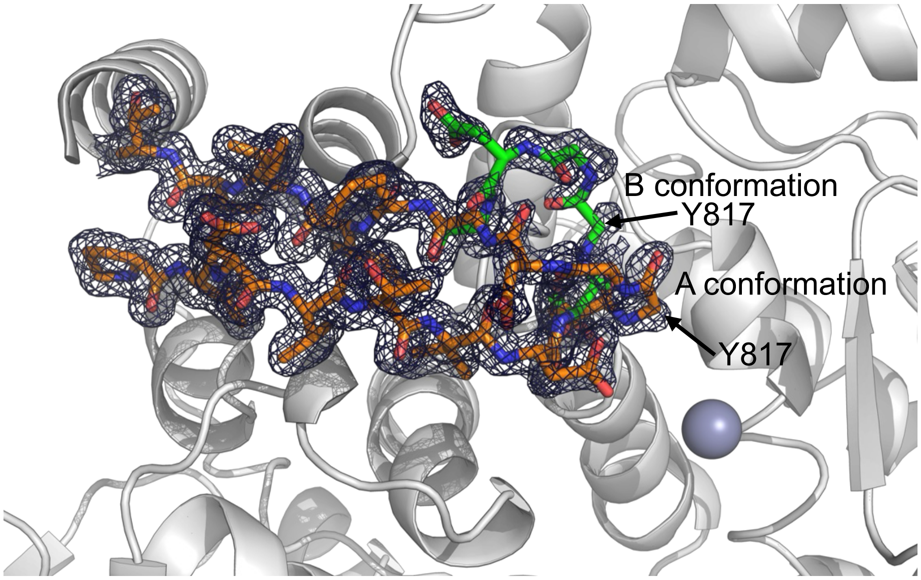

Supplementary Figure 10. Dual conformations of the active site loop in BcM60C. The main conformation is shown as orange sticks and the minor, retracted conformation as green sticks. The $2F_o$-F_c electron density map contoured at 1σ is shown as a mesh.

			P3	P2	P1	P1'	P2'	P3'	P4'			IsoGlycP score	Overall Activity Rating
MUC5AC_R1	D	V	K	P	P	T	T	S	T	T	S	5.5	Good
	P	T	T	S	T	T	S	A	P	P	V	4.0	
MUC5AC_R2	P	V	P	A	T	T	V	G	P	V	P	11.5	Good
MUC5B_R1	D	V	K	G	S	T	A	T	P	S	S	7.4	Good
	K	G	S	T	A	T	P	S	S	T	P	5.0	
	A	T	P	S	S	T	P	G	V	S	K	10.1	
MUC5B_R2	T	T	T	A	T	T	P	T	P	V	S	6.6	Good
	T	A	T	T	P	T	P	V	S	K	G	9.6	
MUC7_R1	T	D	V	K	T	T	A	A	P	P	T	4.0	Good
	T	A	A	P	P	T	P	S	A	T	V	13.3	
	P	T	P	S	A	T	V	S	K	G	E	2.7	
MUC16	V	K	S	V	P	T	T	S	T	P	G	4.5	Good
	K	S	V	P	T	T	S	T	P	G	T	4.8	
	V	P	T	T	S	T	P	G	T	S	-	4.9	
MUC17_R1	D	V	K	S	P	T	N	S	S	P	T	3.9	Good
	T	N	S	S	P	T	T	A	E	V	S	3.8	
MUC1_R1	P	A	P	G	S	T	A	P	P	A	H	16.9	Medium
MUC2_R3	K	S	P	P	P	T	S	T	T	T	L	8.0	Medium
	P	P	T	S	T	T	T	L	P	V	S	2.0	
MUC8_R2	V	H	E	L	P	T	S	S	P	G	G	7.6	Medium
MUC10	T	T	D	S	T	T	P	A	P	T	T	8.4	Medium
MUC11	T	T	P	A	P	T	T	V	S	K	G	8.2	Medium
4/5AC_R2	S	T	G	H	A	T	V	G	P	V	P	4.3	Medium
5AC_R2/4	P	V	P	A	T	T	P	L	P	V	T	8.2	Medium
5AC_R2/11	P	V	P	A	T	T	H	T	T	L	S	1.0	Medium
MUC2_R2	V	K	V	P	P	T	T	T	P	S	P	20.2	Poor
	V	P	P	T	T	T	P	S	P	P	P	16.1	
MUC7_R2	V	K	P	S	A	T	T	P	A	P	P	3.2	Poor
	K	P	S	A	T	T	P	A	P	P	S	8.8	
MUC11/12	S	S	P	G	S	T	H	T	T	L	S	2.3	Poor
ASF	K	A	E	A	P	T	A	V	P	D	K	8.4	Poor
Glycoprotein Iba	P	A	P	S	P	T	T	P	E	P	T	6.6	Poor
	A	P	S	P	T	T	P	E	P	T	V	6.8	
	T	T	P	E	P	T	V	S	K	G	E	5.4	
MUC2_R1	V	K	T	T	V	T	P	T	P	T	P	5.0	Poor
	T	T	V	T	P	T	P	T	P	T	G	46.0	
	V	T	P	T	P	T	P	T	G	V	S	20.5	
	P	T	P	T	P	T	G	V	S	K	G	5.7	
11/5AC_R2	S	S	P	G	S	T	V	G	P	V	P	26.6	Poor
5AC_R2/IgA1	P	V	P	A	T	T	P	S	P	S	P	16.4	Poor
IgA1/5AC_R2	P	S	T	P	P	T	V	G	P	V	V	25.1	Poor
MUC1_R2	K	A	H	G	V	T	S	A	P	D	T	3.3	NA
IgA1_h	P	S	T	P	P	T	P	S	P	S	V	35.9	NA
MUC4	S	T	G	H	A	T	P	L	P	V	T	3.0	NA
MUC3A/B	S	S	I	T	T	T	E	T	T	S	V	1.8	NA
MUC8_R1	P	L	Q	E	G	T	P	G	S	R	V	3.0	NA
MUC19	D	V	K	S	T	T	V	A	P	G	S	5.2	NA
MUC20	V	A	P	G	S	T	T	V	V	S	K	4.3	NA
MUC6_R1	T	T	T	Y	P	T	P	S	H	P	Q	6.9	NA

Supplementary Figure 11. Summarized activity of BcM60K and BcM60C based on the FRET screen analysis. Likelihood of glycosylation was estimated by IsoGlyP with higher scores indicating higher likelihood of being an acceptor site for *O*-glycosylation by ppGalNAcT2.
